# Editorial: Interdisciplinary approaches to antimicrobial use in livestock farming

**DOI:** 10.3389/fvets.2022.971029

**Published:** 2022-08-11

**Authors:** Maria Paula Escobar

**Affiliations:** Bristol Veterinary School, University of Bristol, Bristol, United Kingdom

**Keywords:** antimicrobial resistance, livestock farming, social sciences, interdisciplinarity, complexity

When we first set out to put together a collection that reflected interdisciplinary scholarship on antimicrobial resistance (AMR) in livestock agriculture settings, we wanted to weave together two research threads that seemed to have been running in parallel. The first one, mostly built by veterinarians experimenting with qualitative methods, looks to understand the human contribution to a problem of evolutionary genetics and public health. The second, developed mostly by social scientists, explores the ways in which the AMR problem is framed and what its governance approaches reveal about our ideas of nature, politics, and health. Both strands have continued to grow.

Veterinarians have continued to explore stakeholder perceptions and practises and assess governance landscapes and interventions. Landfried et al. (1) for example, expanded the geographical scope of case studies by looking at veterinarians' prescriptions decisions in Missouri and added goats to the list of species-specific articles. Their study problematises the causal relationship between prescriptions and residue levels as the latter are higher than in other U.S. states even though antibiotics are prescribed in <50% of veterinary visits in Missouri. Meanwhile, Bennani et al. (2) examined the intricacy of UK surveillance systems and revealed inconsistencies in the integration of its 30 key organisations, processes, data sources and working relationships with six international networks.

On the other hand, sociologists Cañada et al. (3) also looked at governance approaches but used ethics as a lens. Their study denounced the intrinsically anthropocentric nature of the public health framing of AMR and argued for alternative ethics that are not founded on human exceptionalism. This interest in how and with what consequences AMR has been framed is recurrent. Hughes et al. (4) for example, take issue with the targets-approach of public and private regulators along the food supply chain and its governing bodies. Similar to Cañada et al., Chandler (5) identifies how the emphasis on individual behaviour change is inconsistent with the connectedness implicit in the One Health approach with which AMR is persistently presented. Chandler shifts the focus from those who prescribe or use antibiotics to antibiotics themselves as materials that harness interspecies relations and make them governable and examines their roles in weaving together the fabric of modern medicine and health systems, and invites us to take seriously the problems that AMR has made visible: our dependence on antibiotics, the risks, and demands of commercial farming and the conditions of increased density in populations, be they animal or human [see also Jamie and Sharples (6)]. This call to understand AMR as a problem that emerges within structural processes of neoliberalisation and the injustices of the global economic system is also made by Dutescu (7) who lays the blame squarely on neoliberalism not only for the emergence of AMR but also for the ineffective behavioural interventions favoured by national and international approaches. Likewise Doron and Broom (8), warn of us of the disproportionate impact of AMR that will deepen global inequalities. Helliwell et al. (9) offer an additional set of warnings, this time related to how the governance of agricultural AMR in the UK places the responsibilities for both the problem and its solution on farmers and veterinarians yet limits their agency when no other elements of the context in which they operate are changed because its concern with animal health is about productivity rather than actual health and welfare.

There are four main ideas that are common to both research strands. First, AMR is a complex problem. Second, dealing with it requires more than behavioural and regulatory approaches because, third, on-farm decisions are not only contingent on context but are also deeply entangled with global and national food systems, agricultural support structures, and veterinary medicine regimes. Thus, and fourth, dealing with AMR calls for a critical and interdisciplinary examination of these systems, structures, and regimes and the inequalities embedded within them. These themes also run through the articles in this Research Topic.

Adam et al. identify external factors that affect transitions to different practices, including the role of slaughterers and distributors as well as that of tangible and intangible objects and materials such as feed and chick quality, vaccines, and alternative medicines. Complexity, they argue, is not limited to the decision to change but extends to the long-term process of transition. Baudoin et al. echo this conclusion and add that success will not only be a matter of long-term support but also of working with temporal and spatial contexts. Doidge et al. add emotions as an additional dimension of complexity, while Hellec et al. bring gender into the picture. Bâtie et al. reveal that in Low and Middle Income Countries this complexity is amplified by structural issues like the number and distribution of veterinarians and their lack of monopoly over the prescription and sale of veterinary medicines. The other two papers looking at LMICs add further elements to this complexity. Jaime et al. step away from behavioural characterisations of AMU as rational, prudent, or responsible and focus instead on the global, national and local agricultural systems through which antibiotics and other veterinary medicines circulate. The logic behind these systems has been transformed from a population health to a market approach where antibiotics have become commodities rather than public goods. In these market economies, Masud et al. elaborate, access appears more determinant than usage attitudes: there is indiscriminate access to too many medicines by too many actors, who at the same time have little access to knowledge and information. Begemann et al. document how market approaches have also led to interventions with unexpected consequences like farmers using residue tests to optimise waste milk management. In turn, Buller et al. tell us that, in their own relationship to the market economy, veterinarians are conflicted about the potential role of diagnostic tools at the farm: farmers making evidence-driven decisions about antibiotic usage is good news but being replaced as figures of authority in health management decisions is not. Redding et al. and Skjølstrup et al. focus too on the farmer-vet relationship, the former looking at the individual factors in play in prescription decisions while the latter adds the external factors to create a model of decision-making complexity.

This Research Topic covers multiple species and locations with a variety of theoretical and methodological approaches that mirror the diverse disciplines and fields the authors represent and the complexity of the problem itself. To deal with this complexity, all the authors agree, inter-and multidisciplinary approaches are essential.

## Author contributions

The author confirms being the sole contributor of this work and has approved it for publication.

## Conflict of interest

The author declares that the research was conducted in the absence of any commercial or financial relationships that could be construed as a potential conflict of interest.

## Publisher's note

All claims expressed in this article are solely those of the authors and do not necessarily represent those of their affiliated organizations, or those of the publisher, the editors and the reviewers. Any product that may be evaluated in this article, or claim that may be made by its manufacturer, is not guaranteed or endorsed by the publisher.

## References

[1] Landfried L Pithua P Lewis RD Rigdon S Jacoby J King CC. Antibiotic use in goats: role of experience and education of Missouri veterinarians. Vet Rec. (2020) 186:349. 10.1136/vr.105455 32079665

[2] Bennani H Cornelsen L Stärk K Häsler B. Characterisation and mapping of the surveillance system for antimicrobial resistance and antimicrobial use in the United Kingdom. Vet Rec. (2021) 188:e10. 10.1002/vetr.10 33835538

[3] Cañada JA Sariola S Butcher A. In critique of anthropocentrism: a more-than-human ethical framework for antimicrobial resistance. Medical Humanities. (2022) 2021:12309. 10.1136/medhum-2021-012309 35321873 PMC9691817

[4] Hughes A Roe A Hocknell S. Food supply chains and the antimicrobial resistance challenge: on the framing, accomplishments and limitations of corporate responsibility. EPA. (2021) 53:1373–90. 10.1177/0308518X211015255

[5] Chandler CIR. Current accounts of antimicrobial resistance: stabilisation, individualisation and antibiotics as infrastructure. Palgrave Commun. (2020) 5:53. 10.1057/s41599-019-0263-4 31157116 PMC6542671

[6] Jamie K Sharples G. The social and material life of antimicrobial clay: exploring antimicrobial resistance, medicine's materiality, and medicines optimization. Front Sociol. (2021) 5:26. 10.3389/fsoc.2020.00026 33869434 PMC8022547

[7] Dutescu IA. The antimicrobial resistance crisis: how neoliberalism helps microbes dodge our drugs. Int J Health Sci. (2021) 51:521–30. 10.1177/0020731420949823 32799748

[8] Doron A Broom A. The spectre of superbugs: waste, structural violence and antimicrobial resistance in India. Worldwide Waste. (2019) 2:1–10. 10.5334/WWWJ.20

[9] Helliwell R Morris C Jones S. Assembling antimicrobial resistance governance in UK animal agriculture. Sociologia Ruralis. (2022) 2022:1–24. 10.1111/soru.12377

